# Medical visual question answering with multimodal: a systematic mini review (2023–2026)

**DOI:** 10.3389/fdgth.2026.1848710

**Published:** 2026-06-12

**Authors:** Maimuna Biswas Noshin, Monoronjon Dutta, Md Nadim Kaysar, Rakib Hossain Sajib, Md Jakir Hossen, Dip Nandi, Abdullah Al Jubair, Mashiour Rahman

**Affiliations:** 1 Department of Electrical and Electronics Engineering, Islamic University of Technology, Gazipur, Bangladesh; 2 ELITE Research Lab, New York, NY, United States; 3 Department of Computer Science and Engineering, Daffodil International University, Dhaka, Bangladesh; 4 Computer Science & Engineering, Nagoya Institute of Technology, Nagoya, Japan; 5 Center for Advanced Analytics (CAA), Faculty of Engineering & Technology (FET), Multimedia University, Melaka, Malaysia; 6 Department of Computer Science, American International University-Bangladesh, Dhaka, Bangladesh

**Keywords:** generative AI in healthcare, large vision-language models, medical visual question answering, multimodal reasoning, systematic review

## Abstract

Medical visual question answering (Med-VQA) has emerged as a critical application of artificial intelligence within a short period of time. Large language models (LLMs) and vision-language models (VLMs) have fundamentally rewritten the architecture of medical question answering (QA). This study aims to systematically analyze recent developments in Med-VQA. Like past methods, which were simple, text-heavy database systems, there has been a shift toward multimodal frameworks. Recent methods are now highly capable of explaining radiology, pathology, and dermatological images along with clinical questions. This review was conducted following PRISMA guidelines, covering 27 representative studies published in various databases, using predefined inclusion and exclusion criteria. The findings reveal a clear shift toward generative models, supported by retrieval mechanisms and structured reasoning strategies such as Chain-of-Thought and multi-agent frameworks. Generative models, along with retrieval-augmented generation (RAG) and preference optimization, are not just more consistent than traditional classification-based methods but also can enable free-form clinical question answering. Though frameworks like multi-agent and hierarchical CoT have significantly improved interpretability and mitigated hallucinations, they also come with some limitations, like higher computational time, multi-view analysis, multi-lingual question answering, lack of standardized evaluation and exploration, domain-specific evaluation, and real-world clinical settings. Med-VQA systems demonstrate significant potential as a clinical decision answer generation with a vision language model. Future work should focus on computational efficiency during real-world validation, fairness evaluation, standardized diagnostic benchmarks, and interpretable reasoning frameworks including specialized domain knowledge and practical skills.

## 1 Introduction

### 1.1 Background

The field of natural language processing (NLP) systems has been substantially transformed by large language models (LLMs). LLMs can now enhance their understanding, text generation, and reasoning abilities when working with text. They can perform in many domains and enhance their ability to make the machines answer questions. In the healthcare domain, LLMs are utilized in many tasks. The task is clinical decision support, diagnostic reasoning, and analyzing biomedical literature to generate relevant responses. Early research work mainly focused on text-based medical question answering. For example, employing textual data to excel in addressing medical questions utilized the Med-PaLM and PMC-LLaMA methods.

However, text is not the only format for medical data. It often includes visual information, including radiology images, pathology slides, and dermatology photographs. There are many difficulties in several types of medical data. One major reason is the limited availability of large and well-annotated datasets. This limitation limits the ability of the applied model to learn effectively from different medical data. As a result, enhancing diagnostic accuracy becomes more difficult. In current studies, multimodal LLMs have been developed using architectures such as CLIP and LLaVA and these models integrate visual encoders with language models. Through this model design, models can perform medical imaging, and they can do so simultaneously. This approach helps the model understand multimodal clinical information more effectively. This progress facilitated research in Medical Visual Question Answering (Med-VQA). In this task, medical images are evaluated, and medically relevant questions are answered by the models.

Swift progress has been observed in Med-VQA from 2023 to 2026. Advanced multimodal systems have replaced traditional text-only approaches in this field. Many recent models include reasoning systems such as chain-of-thought (CoT). The CoT method helps models perform more structured and transparent reasoning. Different methods include retrieval-augmented generation (RAG) and multi-agent collaboration. This RAG and multi-agent collaboration strategy aims to improve the reasoning. Moreover, it improves system capability and enhances prediction accuracy. For the analysis of medical image-text pairs, PubMedCLIP (1) is an example where the CLIP model is optimized for zero-shot Med-VQA performance. Similarly, STLLaVA-Med (2) also uses self-training techniques.

According to our analysis, before and early in 2023, Med-VQA systems were primarily based on transformer-based model architectures where models focused on image-text alignment within fixed vocabularies, limiting responses to predefined answer spaces. By 2024, the field experienced a generative shift, driven by large-scale datasets, along with instruction tuning strategies that enabled models to produce open-ended, context-aware responses. From 2025 to 2026, Med-VQA systems have evolved toward reasoning-driven cognitive thinking enhancement of language models, incorporating multi-agent frameworks. Cognitive thinking is an AI system designed to emulate human-like thinking of language models. In Med-VQA, this allows models to interpret medical images, access relevant knowledge, and perform step-by-step reasoning to produce more reliable answers.

**Figure 1:**
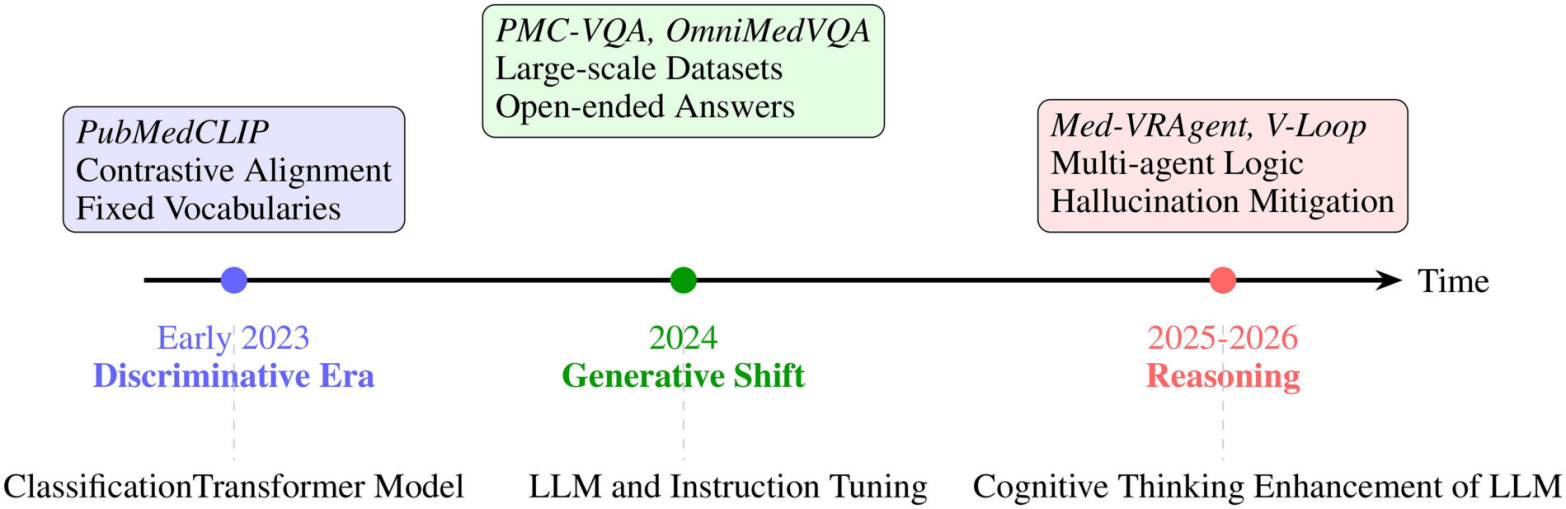
The Med-VQA timeframe (2023–2026) illustrates the transition from discriminative encoders to multi-agent reasoning systems of AI models.

These approaches introduce structured reasoning processes and iterative refinement mechanisms, improving interpretability and reducing hallucinations, thereby moving closer to reliable clinical decision support. These developments show a clear trend toward models that perform well in both zero-shot and few-shot situations, and that can easily adapt to different types of data with minimal changes. Figure 1 shows the latest progress in Med-VQA research.

### 1.2 Statement of the problem

Recent results in this field suggest that LLMs can answer multimodal medical concerns, but certain challenges remain. There are many issues with traditional text-based medical quality assurance methods, including hallucinations, context confusion, and difficulty merging topic-specific medical knowledge. As a result, the output may sometimes be inaccurate. They may also be only partially correct. This issue is very concerning in healthcare settings, and inaccurate responses may affect healthcare decision-making. Moreover, multimodal environments present a significant challenge for visual data. Visual data comes from medical images, such as x-rays and histopathology slides.

Previous studies on radiology and pathology data, such as PathVQA (3) and PMC-VQA (4), highlight the difficulties associated with handling various medical imaging modalities. These challenges occur mainly due to limited datasets. In addition, they require specialized pretraining for medical applications. This limitation can affect how accurately the model interprets associated data. In addition, sometimes, the model fails to correctly identify abnormalities in medical images. It may also be challenging to localize the affected regions accurately. As a result, it reduces the reliability of the output response. In addition, zero-shot performance in medical VQA can be unstable, and the work is between 2023 and 2026. Frameworks improve model interoperability through multi-agent reasoning approaches such as MC-CoT (5) and Med-VRAgent (6). They create many problems during long reasoning processes due to computational cost and error propagation. At the same time, the OmniMedVQA and MEDIQA datasets have become more difficult due to continuous growth. The multilingual questions and open-ended responses method fails to reflect real healthcare conditions. Therefore, there is a clear need for a more unified analysis of existing research to better understand how LLMs can be optimized for both text-based and visual medical question answering.

### 1.3 Significance of the problem

The addition of large language models (LLMs) in multimodal medical question answering (QA) is important for healthcare. Moreover, this is crucial for supporting clinical settings. For making decisions, accurate question-answer systems can assist clinicians. Moreover, an interpretable QA system can work equally well. Moreover, it mitigates the diagnostic cost. For example, for enhancements to both monitoring and treatment, visual QA in dermatology (7) and wound care (8) work perfectly for more precise analysis of skin conditions. On a broader scale, these technologies give access to expert-level medical insights, even in multilingual frameworks (7).

Moreover, the system extends to ethical and regulatory considerations. For example, significant biases and hallucinations must be considered to ensure patient safety. It also maintains trust in medical care assisted by AI. After searching the database, this related work increases from 2023 to 2026. One example of work is MedCoT’s hierarchical expert verification (9), which is an effort to improve reasoning reliability. In a similar way, Towards Top-Down Reasoning (10) developed multi-agent collaboration methods. As a result, this work requires addressing reliability and safety concerns with medical AI systems. There is significant economic potential for using large language models (LLMs) in the healthcare industry. It can significantly reduce operating costs in clinical settings.

### 1.4 Existing studies (findings, limitations)

Zhang et al. (11) proposed a PMC-VQA dataset that contains 149k medical images with 227k question pairs. Their study secures 16% accuracy from training with domain-specific pretraining. Knowledge-guided alignment has emerged as a powerful technique for improving model performance in clinical report generation. Yin et al. (12) developed a KIA framework aimed at incorporating clinical knowledge into the analysis of chest x-rays. This method improves both the accuracy and clinical relevance of the resulting reports. In a similar context, Luo et al. (13) developed a multimodal model that highlights anatomical structures and abnormalities in both 2D and 3D medical imaging. Their study on visual grounding analysis demonstrated a 4.1% increase in VQA performance. Prompt-based methods have notably enhanced interpretability and reduced annotation costs, especially in medical imaging tasks where precise labeling is essential for effective model training, such as in the detection of abnormalities in CT, MRI, and x-ray images. Zhu et al. (14) utilized multiple datasets, including SLAKE, VQA-RAD, and PMC-VQA. Their work focuses on detecting abnormalities using CT, MRI, and x-ray. Ultimately, their work attained over 10% accuracy enhancement on VQA-RAD. Hein et al. (15) showed the CheXalign model, a fine-tuning approach that corresponds model outputs with clinical expectations without demanding extensive human feedback, further diminishing reliance on annotated datasets.

Med-VQA system work helps for detecting and diminishing hallucinations. For example, Jin et al. (16) developed a V-Loop model that helps logical verification of visual reasoning loops. Their method helps effectively detect hallucinations. Ma et al. (17) demonstrated that LLM-improved data mining supports precise reasoning in VQA tasks. This underscores the potential for integrating large pre-trained models with domain-specific expertise. Furthermore, other researchers applied Med-VQA to several datasets involved, such as chest x-rays. For example, Yilmaz et al. (18) applied a method of clinical decision-making using dermatology data. Nagao et al. (19) employed chest CT scans. Similarly, Zhu et al. (20) excel in their work through VQA methods. For screening and detecting breast cancer, they introduced MammoVQA. Their developed method improves accuracy regarding the decision-making process that is involved in diagnoses. Ultimately, this analysis results in better patients and more effective treatment plans. For medical AI uses, explainability plays a significant role. For example, Gai et al. (21) developed a framework, MedThink, which offers both answers and textual explanations. This method improves the interpretability.

This analysis identified multiple problems. The availability of annotated medical datasets is one of them. When a model utilizes one dataset, it does not show perfect results in healthcare. Furthermore, hallucinations are one of the critical issues in healthcare. This issue helps create incorrect and misleading responses. To overcome these issues, we require more robust and reliable methods.

### 1.5 Motivation of the study

This analysis, spanning 2023 to 2026, highlights the necessity for thorough synthesis. Numerous contributions have emerged in Med-VQA in this period. For example, “From Images to Textual Prompts” (22) and “Multimodal Prompt Retrieval” (23) have achieved significant progress in zero-shot capabilities. However, this systematic analysis is incomplete. This analysis summarizes current research trends and new methods. The literature is rapidly advancing from discriminative models to generative structures with human-like reasoning processes (10). Through the evaluation, common issues were identified, possible research directions were suggested, and future efforts in rapid shifts were guided. Further, the development of new datasets and benchmarks has further complicated this work. For example, the PMC-VQA (4) and the OmniMedVQA (24) demonstrate the expanding scope of Med-VQA research; both works included 12 medical methods. These advances provide new challenges, including domain adaptation, analysis consistency, and medical ethics. In this review paper, we aim to analyze text- and image-based Med-VQA research studies to provide a clear overview of the different system architectures, reasoning process, and approaches that contribute to Med-VQA, as well as the integration of LLMs into healthcare decision-support tools. This review is intended to help future researchers identify existing research limitations, recent trends of VQA research and explore promising future research directions.

### 1.6 Objectives of the study

This mini-review aims to analyze the recent trends in medical visual question answering systematically by analyzing model architectures, reasoning strategies, datasets, and evaluation practices in order to identify methodological trends, limitations and future research directions. The primary objectives of this review are:

1. We will systematically analyze LLM-based architectures for medical question answering (QA) from 2023 to 2026 emphasizing architectures, datasets, and assessment criteria for both text-only and multimodal approaches.
2. Examine architectural innovations, including CoT frameworks like MedCoT and MC-CoT (5, 9), retrieval systems such as MasonNLP’s RAG (8), and agent-based systems like Med-VRAgent, and assess their robustness.
3. The analysis aims to showcase zero-shot flexibility and linguistic support, while also shedding light on limitations, data dependence, computing loads, and hallucinations.
4. We will examine how incorporating advanced reasoning frameworks can ensure the reliability and clinical viability of a multimodal medical QA system.

### 1.7 Research questions

According to our analysis objectives, the reviewed papers demonstrate significant advances in the Med-VQA field; however, our analysis also identifies several critical gaps. These gaps are guided by a few research questions for future researchers to investigate, providing a structured synthesis of architectural, methodological, and evaluation trends in Med-VQA :

1. How do advanced generative multimodal reasoning mechanisms influence cross-lingual transfer, diagnostic accuracy, and robustness of Med-VQA architectures under low-resource language constraints?
2. What reasoning and retrieval strategies (e.g., Chain-of-Thought, multi-agent systems, RAG) have been proposed in Med-VQA and how do they impact interpretability, hallucination mitigation, and performance?
3. How do real-world MRI or x-ray specialists reason through clinical cases using domain knowledge and practical expertise, and how can these reasoning processes be computationally modeled—using a multi-agent architecture—to enhance the effectiveness and reliability of AI-based Med-VQA?
4. How effective is the multiple view of the medical image for answer generation with different types of reasoning processes in system architecture?
5. How are datasets and evaluation metrics used across studies for model improvement and to what extent do they enable consistent and reliable comparison of Med-VQA models?
6. What are the key technical and practical challenges in deploying these models in real-world clinical Med-VQA systems, including computational efficiency, generalization, computational time, and clinical validation, and how can these limitations be effectively overcome?

Collectively, the research questions ensure the review addresses architectures, reasoning techniques, evaluation metrics, and deployment challenges in Med-VQA.

## 2 Methodology

### 2.1 Study design, data sources, participants

This review paper has built on methodical literature review architecture to converge studies on LLMs applied to medical QA. Being conducted in a scholarly setting, this paper utilizes digital libraries and databases. Our search strategy utilizes the most influential repositories in computer science, artificial intelligence, and biomedical informatics, like ACM Digital Library, IEEE Xplore, PubMed, arXiv, Scopus and Google Scholar. Priority was given to English-language publications with no geographical constraints. From an initial pool of hundreds of papers, we incorporated 27 papers (as summarized in Table 2). We focused on methods and frameworks used.

### 2.2 Literature search strategy

To identify appropriate studies, a comprehensive literature search was carried out. To construct a top-tier overview of the current landscape, we researched through some keywords, including “LLM, Med-QA, multimodal, visual question answering”. Moreover, to filter out past research works, publication date was selected from 2023 to 2026. To keep the relevancy supreme, document types were articles, proceedings, and preprints. Any kind of gray literature was excluded to maintain focus on peer-reviewed contributions.

### 2.3 Inclusion and exclusion criteria

For ensuring the relevance and grade of the selected studies, predefined standards are used for inclusion during the screening process. Papers are selected when they focus on visual question answering (VQA) in medical imaging and use multimodal approaches combining visual and textual data. Further scrutiny was used to find papers with a clear methodology, including model design, dataset description, and experimental evaluation with quantitative implementation metrics. Studies are excluded when they did not employ multimodal architectures, affect medical imaging, address the VQA task or were only text-based medical question answering without visual input. Duplicate papers were also eliminated.

### 2.4 Study selection based on PRISMA

**Figure 2:**
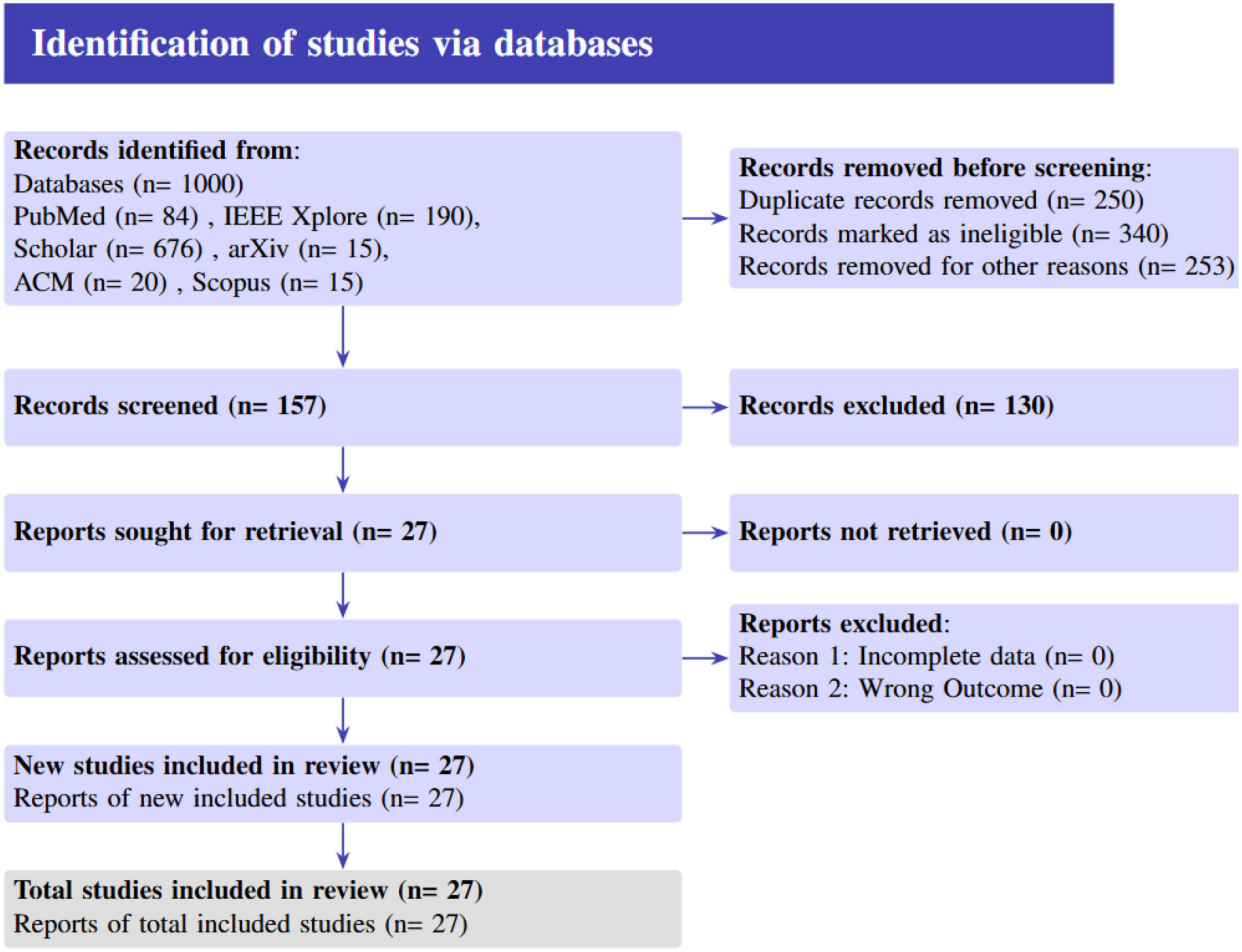
PRISMA flow diagram.

We followed the Preferred Reporting Items for Systematic Reviews and Meta-Analyses (PRISMA) guidelines to guarantee the integrity and transparency of the selection process. This is illustrated in Figure 2, detailing the identification, screening, eligibility, and inclusion stages. To maintain the quality and relevancy, we primarily selected 1,000 papers at first based on titles and abstracts. We resolved any discrepancies through consensus to confirm that only the robust, relevant papers are selected. Ultimately, a final set of 27 papers was selected, which represent the key themes like zero-shot Med-VQA [e.g., STLLaVA-Med (2)], multi-agent reasoning [e.g., MedCoT (9)], and benchmark development [e.g., OmniMedVQA (24)].

### 2.5 Data extraction and analysis

A standardized table template was used to conduct the data extraction and analysis. The main focus was on author(s), focus, methodology (e.g., CoT, RAG), datasets (e.g., VQA-RAD, SLAKE), key results (e.g., precision, contribution), evaluation metrics (e.g., BLEU, ROUGE), advantages (e.g., zero-shot adaptability), and limitations (e.g., hallucinations). By grouping the papers into three parts: text-based QA inventions, multimodal visual enhancements, and hybrid methods. The shift toward generative models [e.g., PMC-VQA (4)] and multi-agent frameworks [e.g., MC-CoT (5)]—these narrative syntheses were used to ensure a balanced, evidence-based synthesis of the literature.

## 3 Literature review and thematic analysis

### 3.1 Theme 1: transition from discriminative to generative and open-ended Med-VQA approaches

The years between 2023 and 2026 draw a precise paradigm of shift in Med-VQA. The trend shifted from discriminative, closed-set classification models towards generative, open-ended architectures. In the early stages, influential works such as PubMedCLIP (1) and PathVQA (3) laid the groundwork by relying on pre-training and classic VQA pipelines (e.g., VisualBERT-style encoders). This method enhanced the visual experience on relatively small, domain-specific datasets (VQA-RAD, SLAKE, PathVQA). Though these methods achieved moderate accuracy gains (nearly 3%–6%), they faced problems like fixed answer vocabularies and lack of open-ended questions. PMC-VQA (4), which introduced a large-scale (227k QA pairs, 149k images) instruction-tuning dataset, marked a transition toward generative modeling. This study showed that generative open-ended Med-VQA could reach new state-of-the-art results on multiple benchmarks by aligning a strong vision backbone (PMC-CLIP) with LLaMA-based language decoders.

This model can also enable free-form clinical dialogue. This accelerated the transition from classification heads toward text generation, including STLLaVA-Med (2), which employed self-training with Direct Preference Optimization (DPO) and achieved competitive zero-shot performance using only 9% of conventional medical training data. By incorporating multimodal prompt retrieval (23) with modality-independent generative models, the evolution continued. It encourages rapid few-shot/zero-shot domain fine-tuning (up to 30% accuracy) and instruction-tuning modes that employ frozen large language models with synthetic visual prompts (22).

Frameworks like MedCoT (9), MC-CoT (5), Med-VRAgent (6), and MasonNLP’s RAG-enhanced system (8) proved that there has been a significant advancement in the Med-QA field. Moving beyond simple models, these models generate free-text responses more like a clinician. Not only that, these models also try to eradicate hallucinations via reasoning chains, retrieval, or multi-agent verification.

### 3.2 Theme 2: reasoning enhancement strategies: chain-of-thought, multi-agent collaboration, self-reflection

For boosting both precision and diagnostic transparency, emphasis has been given to detailed reasoning mechanisms. Frameworks like MedCoT (9) and MC-CoT (5) were highly adapted to Med-VQA through Chain-of-Thought (CoT) prompting. The systems do not just diagnose but utilize modular collaborative reasoning, employing multi-turn LLM-MLLM. In particular, MedCoT (9) shows consequent hallucination deduction via self-reflection. Towards Top-Down Reasoning (10) uses three agents collectively to make a multi-view knowledge base and clarify responses. Furthermore, to mitigate hallucination, Med-VRAgent (6) works with Visual Guidance, Monte Carlo Tree Search (MCTS), and Proximal Policy Optimization (PPO) fine-tuning in the field of radiology and pathology images.

### 3.3 Theme 3: benchmarking and domain-specific adaptation

The recent addition in this field is the rapid expansion of evaluation resources. Earlier, researchers mostly relied on datasets (VQA-RAD, SLAKE, PathVQA), which were limited in scale and modality coverage. With passing time, PMC-VQA (4) (227k QA pairs) and OmniMedVQA (24) changed this landscape. With poor image alignment, OmniMedVQA presented critical weaknesses in the existing LVLMs. MEDIQA-WV (wound care) (8) and MEDIQA-M3G (7) use shared-task datasets thus advancing multilingual evaluation. This scenario shows the urgent need for clinically realistic testing environments.

**Figure 3:**
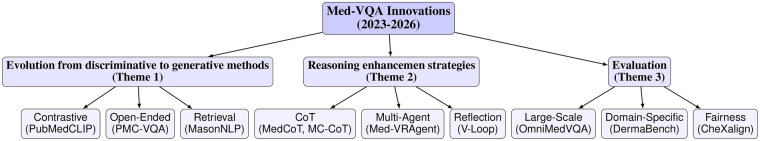
Categorization of Med-VQA research.

Figure 3 demonstrates the thematic analysis of the studied papers.

### 3.4 Comparative analysis

The analysis of the studied papers reveals a technological hierarchy. Those models combining domain-specific pre-training (PMC-CLIP, PubMedCLIP) with generative decoding are the most successful systems (1, 2, 4). In few-shot domain adaptation, retrieval-augmented and prompt-retrieval methods excel (8, 23). On the other hand, pure generative models face poor generalization without retrieval. At the cost of high computational cost, multi-agent and CoT frameworks provide the strongest interpretability and hallucination reduction (4%–10% gains) (5, 6, 9, 10). Also, technological benchmarks reveal continuous lacking in multi-modality range and multi-step reasoning (20, 24). To ensure fair comparison, Med-VQA studies should be interpreted within task-specific categories, including classification-based VQA, open-ended answer generation, and visual grounding. Each category relies on distinct evaluation metrics.

### 3.5 Strengths and limitations of the reviewed papers

**Strengths**

- The shift toward data-efficient and zero/few-shot learning with the help of self-training, methodical prompts, and retrieval (2, 22, 23)
- By integrating CoT, agent collaboration, and verification, there has been improvement in interpretability (6, 9, 10).
- Open-ended clinical utility with free-form along with multilingual generation (4, 7).
- Large-scale realistic benchmarking (8, 24).
- Active hallucination mitigation with the use of reflection and external knowledge (16, 17).

**Limitations**

- Continuous hallucinations, mostly in long reasoning chains (5, 9).
- High inference cost of multi-agent/MCTS methods (6)
- Lack of standardized evaluation for task-specific datasets.
- Sensitivity to retrieval quality, prompt design, and base-model biases (7, 8).
- Limited modality generalization and multi-step clinical reasoning (24).
- Insufficient real-world prospective validation.

### 3.6 Literature gaps

Though there have been several works on standardized benchmarks, an absence of standardized multi-step diagnostic workflow benchmarks can also be found. As most of the models focus on only one language, especially on English, multilingual, low-resource, and fairness-aware settings are under-explored. The prototype run and real-world prospective clinical scenarios are completely different. There remains a gap in running pilot projects in real clinical settings. While most of the medical images require multi-view images to get the overall aspect of any disease, there is insufficient handling of temporal/multi-view imaging series in the literature. Some models can be found with high explainability, but they often come with high computational cost. These gaps delineate valuable directions for future research toward clinically viable, trustworthy, and equitable multimodal medical QA systems.

## 4 Results

### 4.1 Our key findings

This systematic mini-review examines the field of medical question answering, focusing on the application of LLMs and VLMs. The comparative performance of various models across medical VQA benchmarks is summarized in Table 1, which distinguishes between closed-ended (Table 1a) and open-ended (Table 1b) evaluation paradigms.

**Table 1:** Performance comparison.

Paper	Clinical domain	Metric	Performance
(a) Closed-ended Med-VQA			
Wang et al. (25)	Multidisciplinary	Accuracy	90.77% on SLAKE
Wang et al. (10)	General & Radiology	Accuracy	68.6% on A-OKVQA
Zhang et al. (4)	Multi-domain Medical	Accuracy	40.2% (MedVInT-TE)
Ossowski and Hu (23)	Multimodal Medical	Accuracy	30% gain
Eslami et al. (1)	Multi-domain Medical	Accuracy	3% gain over SOTA
Liu et al. (9)	Multi-domain Medical	Accuracy	87.50% on VQA-RAD
Luo et al. (13)	Multi-domain	Accuracy	87.3% on SLAKE
Zhang et al. (11)	Multi-domain (RadFM)	Accuracy	58.8% on VQA-RAD
Wang et al. (26)	Chest x-Ray (Radiology)	Accuracy	76.3% on MIMIC–Diff–VQA
Guo et al. (6)	Multi-domain	Accuracy	46.74% on GMAI-MMBench
		Metric	
Paper	Clinical domain	BLEU	ROUGE
(b) Open-ended Med-VQA			
Liu et al. (9)	Multi-domain (SLAKE)	78.33 (B-1)	80.86 (R-L)
Liu et al. (9)	Multi-domain (VQA-RAD)	61.29 (B-1)	66.30 (R-1)
Gai et al. (21)	Multi-domain (SLAKE)	77.94 (B-1)	80.12 (R-1)
Luo et al. (13)	Multi-domain (VividMed)	88.0 (B-1)	88.5 (R-1)
Yin et al. (12)	Chest x-Ray (KIA)	50.3 (B-1)	38.5 (R-L)
Wang et al. (26)	Chest x-Ray (Radiology)	50.0 (B-4)	65.5 (L)
Guo et al. (6)	Radiology	33.45	26.81 (L)

It is important to note that performance across Med-VQA studies is evaluated using diverse metrics depending on task type. Classification-based tasks typically use accuracy and F1-score, while generative tasks rely on BLEU, ROUGE, CIDEr, and BERTScore. Therefore, direct comparison using a single metric may not fully capture model performance across heterogeneous tasks. The key findings from the papers are:

#### 4.1.1 Generative open-ended models have become the fundamental shift

The shift led by frameworks like PMC-VQA (4) and STLLaVA-Med (2) has fundamentally changed the perspective. On open-ended clinical tasks, generative approaches consistently outperform traditional classification-based methods, (achieving 5%–16% gains in accuracy in classification settings), while also showing superior results in generative evaluation metrics such as BLEU and ROUGE. Along with this, it also enables free-form diagnostic dialogue.

#### 4.1.2 Reasoning enhancement methods significantly improve interpretability

Through multi-agent collaboration (6, 10), hierarchical Chain-of-Thought (CoT) (9), and self-reflection loops (5, 16), modern models have achieved gain in factual consistency(e.g., SMR-Agents reaching 90.77% accuracy on SLAKE). These approaches help to reduce hallucinations. With the mitigation of hallucination, trustworthiness increases. This process also ensures that the models are not only accurate but also that the answers have been reached through a logical path.

#### 4.1.3 Retrieval-augmented and prompt-retrieval methods are better in data efficiency

Methods like leveraging multimodal retrieval (FAISS, CLIP-based KNN) (8, 23) and synthetic instruction data (2, 22) prove that we do not always need massive, annotated datasets, as these models also achieve rapid zero-shot/few-shot adaptation (up to 30% accuracy gains).

#### 4.1.4 Large-scale benchmarks expose persistent weaknesses

While individual models show high performance on specific datasets, comprehensive benchmarks like OmniMedVQA (24) reveal a performance gap across diverse modalities. Experiments show that even specialized Med-VQA models struggle with real-world medical images across 73 different datasets, highlighting the need for better fine-grained image alignment.

#### 4.1.5 Domain-specific adaptation yields better gains

Tailored models for dedicated fields like dermatology (7), wound care (8), mammography (20), and pulmonary imaging (19)—they are the evidence that domain-specific models show better localization along with fairness evaluation. It can be said that while throughout the years, significant progress has been made toward clinically viable multimodal medical QA, there are also challenges like hallucination, computational cost, and insufficient real-world validation, which have to be addressed in the future.

### 4.2 Multi-modal visual question answering

In Table 2, we have summarized the multi-modal visual question-answering method, its limitations, and other details.

**Table 2:** Overview of LLM and generative AI studies in visual question answering and healthcare.

Author(s)	Focus	Multi-model	Dataset	LLM/NLP method	Task/objective	Key findings	Evaluation metric	Advantages of LLM	Limitations/challenges
Zhang et al. (11)	General medical visual understanding	Multi-model	PMC-VQA	MedVInT (PMC-CLIP + LLaMA-7B / GPT-4, prompt-based)	MedVInT(LLaMA-7B, PMC-LLaMA, PubMedBERT, and GPT-4)	Achieves ∼16% accuracy gain over training from scratch via domain-specific pretraining	Accuracy, BLEU-1, AUC	Scalable and flexible reasoning	Bias and hallucinations
Yin et al. (12)	Chest x-ray clinical abnormality analysis	Multi-model	IU-Xray, MIMIC-CXR	BERT encoder(text) + ViT(image) + Transformer decoder(text)	Automated radiology report generation	Knowledge-guided masking improves fluency and clinical accuracy	BLEU, ROUGE, CIDEr, CE, Precision, recall, and f1-score metrics	Efficient and reduced model size	Domain-specific (CXR only)
Luo et al. (13)	Anatomical structures and abnormalities in 2D and 3D medical imaging	Multi-model	MIMIC-CXR, CT-RATE	CogVLM (Vicuna 7B) + rsLoRA + SAM grounding	Detection, localization, report generation	Visual grounding improves VQA accuracy by 4.1%	BLEU, ROUGE-L, CheXpert F1, Dice	Easily verify AI predictions against medical images	Scarcity of grounded annotations
Zhu et al. (14)	Detecting abnormalities using CT, MRI, and x-ray	Multi-model	SLAKE, VQA-RAD, PMC-VQA	ViP-LLaVA 7B + Grounding DINO	Region-focused VQA	Over 10% accuracy improvement on VQA-RAD	Accuracy, Recall	Explainable region highlighting	Prompt sensitivity
Hein et al. (15)	Radiology Report Generation (RRG) and CXR Interpretation	Multi-model	MIMIC-CXR, CheXpert	Direct Alignment Algorithms (DPO, IPO, LC-DPO)	Factually grounded report generation	CheXbert score improves by 7%–14%	GREEN, LC-GREEN, CheXbert F1	Scalability and Efficiency	Reward over-optimization
Ma et al. (17)	Medical visual and linguistic information question answering	Multi-model	VQA-RAD, SLAKE, MED-VQA	Latent Knowledge Prompt Generation (LLM-based)	Generate responses for both open-ended and closed-ended medical queries	Achieves 79.2% accuracy on VQA-RAD	Accuracy	Efficient intention-aware reasoning	Medical data scarcity
Jin et al. (16)	Hallucination detection in Med-VQA	Multi-model	VQA-RAD, VQA-Med-2019, SLAKE	Training-free bidirectional reasoning	Answer verification	Effective hallucination detection in complex QA	AUC, AUG	Scalable and interpretable	Depends on base model strength
Yilmaz et al. (18)	Dermatology fairness and reasoning	VLMs	DDI (656 clinical images)	Prompt-based VLM benchmarking	Clinical reasoning and fairness audit	Clinician-curated dataset improves reliability	Qualitative + VQA metrics	Fairness-aware evaluation	Dataset limitation
Nagao et al. (19)	Pulmonary nodule morphology	Multi-model	LIDC-IDRI	Fine-tuned BLIP (prompt-based)	Interactive image finding generation	Outperforms prior BLIP models	BLEU, CIDEr, MAE	Interpretable and interactive	required improve performance
Butsanets et al. (27)	Multi-organ radiology reasoning	Multi-model	RadImageNet-derived dataset	Fine-tuned prompt-based VLMs	Structured reasoning assessment	Pathology detection remains a bottleneck	Mean Accuracy	Grounded reasoning	Shortcut vulnerability
Zhu et al. (20)	Breast cancer mammography	Multi-model	MammoVQA (15 datasets combined)	LVLM + LoRA fine-tuning	Breast cancer VQA	Achieves +21% external validation accuracy	Accuracy, Macro-F1	Strong generalization	Class imbalance
Gai et al. (21)	Explainable Med-VQA	Multi-model	R-RAD, R-SLAKE, R-Path	MedThink (T5-based, CoT reasoning)	Rationale-based QA	SOTA accuracy with 1/10th parameters	Accuracy, BLEU, ROUGE	Cost-effective interpretability	Hallucination risk
Sun et al. (2)	LVLM	Multi-model	Med-60k-IM, auto-generated preference data	GPT-4o, policy LVLM fine-tuned with DPO	Medical VQA (open and closed)	Achieves competitive zero-shot performance using 9% of data, stronger reasoning with less data	Recall and F1, Accuracy, SLAKE, PathVQA	Massive data efficiency (9% data), reduces expensive medical annotation	Quality depends on GPT-4o
Eslami et al. (1)	MedVQA	Multi-model	ROCO, VQA-RAD, SLAKE	Fine-tuned version of CLIP	Improves the visual encoder for MedVQA via medical contrastive pre-training	Boosts downstream MedVQA accuracy by up to 3% absolute over previous SOTA	Accuracy on VQA-RAD, SLAKE	Leverages huge unannotated PubMed image-text pairs	Text encoder weaker than vision encoder for questions
He et al. (3)	Image and text VQA	Multi-model	PathVQA, QA pairs from textbooks, PEIR digital library	Traditional VQA (VisualBERT-style)	Pathology VQA	3-level opt framework removes noisy SSL examples—5.8% accuracy	Accuracy, BLEU, F1	Handles extreme data scarcity, automatic noise removal in SSL	Noisy self-supervision
Ossowski and Hu (23)	Domain-agnostic generative model	Multi-model	SLAKE, VQA-RAD	Multimodal prompt retrieval (CLIP embeddings for KNN of image-question pairs)	Open/closed-answer VQA generation	Retrieval boosts domain adaptation by up to 30% accuracy	Accuracy, SLAKE- VQA-RAD	Rapid zero/few-shot adaptation to new distributions, mitigates catastrophic forgetting in fine-tuning	Weak on complex reasoning
Liu et al. (9)	Interpretable Med-VQA via hierarchical expert verification	Multi-expert	VQA-RAD, SLAKE-EN, Med-VQA, PathVQA	Gemini Pro, Flan-T5, sparse MoE	Generate answers and transparent reasoning paths	Sparse MoE outperforms single-model SoTA and self-reflection reduces hallucinations	Accuracy, ROUGE/BLEU	Enables verifiable reasoning chains, hierarchical structure improves robustness	Still susceptible to LLM hallucinations
Karim and Uzuner (8)	Wound-care	Multi-model	MEDIQA-WV	LLM with FAISS-based multimodal retrieval	Generation and structured extraction	Achieves good performance without domain training, reduces hallucinations	dBLEU, ROUGE, BERTScore	Enables open-ended generation and multilingual support, integrates visual features for context-based answers	Mirror retrieval data biases, sensitive to irrelevant neighbors
Saeed (7)	Dermatology	Multi-model	MEDIQA-M3G dermatology dataset	Pre-trained QA models and CLIP contrastive learning	Generate insightful answers to open-ended questions in multiple languages	Achieves high rankings in the shared task; contrastive learning helps uncertainty quantification	DeltaBLEU and BERTScore	Significant efficiency, high interpretability	Performance depends on the quality of pre-trained QA models
Hu et al. (24)	Med-VQA	No agents, benchmark evaluates 12 LVLMs	73 public medical classification datasets	GPT-3.5 for QA pair generation and option creation	Benchmarking LVLMs on medical VQA	Highlights the need for better medical image-text alignment	Question-answering score, Prefix-based score	Reducing manual annotation cost while creating a realistic, large-scale benchmark	Potential dataset biases from source classification tasks
Guo et al. (22)	Visual instruction tuning	Multi-modal	VQAv2, OK-VQA, A-OKVQA	Frozen OPT, GPT-J, BLOOM, synthetic QA prompt construction	Zero-shot open-ended VQA	Synthetic QA pairs are more effective than plain captions, scaling LLM size gives clear gains	Standard VQA accuracy	Low deployment cost	Quality of synthetic QA pairs depends on the captioner and question generator
Zhang et al. (4)	Zero-shot VQA	Multi-modal	PMC-VQA	PMC-LLaMA / LLaMA + PMC-CLIP vision backbone	Generative MedVQA	MedVInT-TE and PMC-CLIP reaches new SOTA	Accuracy + BLEU-1	Strong scaling with domain-specific backbones enables free-form clinical dialogue	Lacks uncertainty quantification
Wang et al. (10)	Top-down clinical reasoning like human	Multi-agent	ScienceQA, A-OKVQA, VQA-RAD, Winoground	GPT-4o-mini, LLaVA, multi-agent prompt engineering	Explainable zero-shot VQA along with top-down reasoning	Confidence word dramatically improves interpretability	Accuracy, manual interpretability survey	High interpretability, continuous improvement with better LLMs, error mitigation	Error in long agent chains, prompt-heavy
Wei et al. (5)	Zero-shot Med-VQA	Multi-agent	SLAKE, VQA-RAD, PATH-VQA	GPT-3.5, LLaVA, DeepSeek-VL , Qwen-VL (MLLM)	Zero-shot open-ended Med-VQA (generative)	Outperforms standalone MLLMs and other CoT frameworks in accuracy and recall	Recall of key information, LLM-as-a-judge accuracy	Zero-shot scalability, no task-specific fine-tuning, strong interpretability via explicit modules and hypotheses	Heavy prompt engineering dependence, potential error propagation in long CoT chains
Guo et al. (6)	Radiology and pathology	Multimodal agent framework	EHR-derived medical images (IU-Xray, MIMIC-CXR), VQA-RAD, GMAI-MMbench	VLMs, multi-agent, MCTS, PPO fine-tuning	New SOTA performance, superiority over baselines	Reduces hallucinations and improves localization and spatial reasoning	Accuracy, BLEU, ROUGE-L	Reduces hallucinations and improves localization and spatial reasoning	Computationally heavy (MCTS), limited generalization beyond trained domains
Wang et al. (26)	Chest x-ray (Radiology)	Knowledge-enhanced follow-up framework	MIMIC-CXR, Open-I	VLM, Domain Knowledge Retrieval	Highest accuracy in follow-up VQA tasks	Captures temporal disease progression via domain-specific knowledge	Accuracy, BLEU, ROUGE-L, CIDEr	Effective temporal reasoning and reduction in clinical hallucinations	Specific to Chest x-rays
Wang et al. (25)	Radiology, Pathology	Multimodal	VQA-RAD, SLAKE, Path-VQA	MLLM, Multi-agent Collaboration	SOTA zero-shot performance across diverse datasets	Simulate multidisciplinary consultation for high interpretability	Accuracy, F1-score, BLEU, ROUGE	Strong zero-shot generalization and transparent, traceable reasoning paths	Sensitive to initial MSG quality

## 5 Discussion

### 5.1 Contextual interpretation

The evolution in the architecture represents that it has not been done overnight but the broader trends in foundation models. Models like LLaMA, GPT-4, and LLaVA ensure the forward move in general-domain vision-language tasks with instruction tuning and generative decoding. Just like real-world clinical scenarios, recent models emphasize explicit reasoning and multi-agent verification for diagnosis and iterative refinement. Like this, the rapid growth of large-scale benchmarks (PMC-VQA, OmniMedVQA) and shared tasks (MEDIQA series) presents increasing community coordination within the field. From clinical perspective, the ability to produce free-form, multilingual, and contextually grounded responses (7, 8) has the potential to support telemedicine, second-opinion systems, and patient education. However, the continued presence of hallucinations—even in advanced reasoning frameworks—shows that current models remain assistive in real clinical settings.

### 5.2 Compare and contrast

Looking back to the pre-2023 era, the methods faced problems, such as the training sets being narrow. So models faced problems with anything outside of the training set, and the systems were rigid. After that period, generative approaches have mitigated those barriers and offer greater flexibility and clinical utility. But they often face higher hallucination risk and inference cost and time. Then retrieval-augmented methods (8, 23) come into scenario and outperform generative baselines in domain-shift scenarios. Still, they remain sensitive to retrieval corpus quality and irrelevant neighbor effects. Multi-agent and CoT frameworks (6, 9, 10) show enhancement in interpretability and consistency. Compared to single-model generative methods these increase the clinical safety. But they also suffer from higher computational overhead (MCTS, multi-turn interaction) due to iterative inference, multiple model interactions, and increased token generation, increasing the computational cost and time. This added complexity can lead to higher resource consumption compared to conventional single-pass models. In real-world clinical practices, where rapid decision-making is often critical (e.g., emergency radiology workflows or ICU settings), such latency may hinder real-time deployment. While high-resource clinical environments may better accommodate these computational demands, latency and efficiency remain important considerations across all healthcare settings. Additionally, resource-constrained healthcare systems, particularly in low- and middle-income regions, may face challenges in supporting the computational requirements of these advanced models. There exists a trade-off between interpretability and deployability, highlighting the need for efficient, optimized architectures A parallel challenge lies in the divide between specialized (dermatology, mammography) and generalist benchmarks(OmniMedVQA). Specialized benchmarks obtain higher task-specific interpretation. Ultimately, no single method is dominant now. Rather, hybrid architectures show better performance regarding incorporating retrieval, reasoning chains, and domain-specific pre-training, increasing interpretability and efficiency.

### 5.3 Limitations of this review

This review has a few limitations to be acknowledged. The study is restricted to publications from 2023 to 2026, which, while capturing recent advancements, may exclude earlier foundational work. Only English-language publications were considered. Although multiple databases (ACM, IEEE, Scopus, arXiv, Google Scholar) were used, relevant studies outside these sources may have been missed. Finally, the final analysis includes 27 representative papers, which may not fully capture the rapidly evolving Med-VQA landscape.

### 5.4 Recommendation and future work

To advance the Med-VQA system, future research must emphasize the development of standardized multi-step diagnostic benchmarks. The systems should have the ability to simulate real clinical reasoning workflows (sequential imaging, temporal series, and multi-side view reasoning). While current frameworks show computational efficiency, reliable explainability, in future methods that can preserve high interpretability without MCTS-level overhead should be explored. Prospective clinical validation studies should be carried on for evaluating the model’s impact on diagnostic accuracy, time of decision, and patient outcomes, multilingual datasets. Besides, to address global medical deficiencies, efforts should be given on fair datasets and evaluation benchmarks. To reduce hallucination, external structured knowledge bases have to be integrated. Finally, we recommend future researchers to investigate research questions of 4.4 section.

### 5.5 Conclusions

This systematic review synthesizes recent advancement in the Med-VQA landscape, highlighting a fundamental transformation from classification-based systems to generative, reasoning-enhanced multimodal architectures. The significance of this review lies in providing a structured synthesis of architectural trends, reasoning mechanisms, datasets, and evaluation practices within the rapidly evolving Med-VQA domain. Across recent developments, models increasingly integrate visual understanding with language generation, supported by retrieval mechanisms and structured reasoning strategies, enabling more flexible and clinically relevant responses. Several key patterns emerge from this evolution. First, generative models consistently outperform earlier approaches. Second, methods such as hierarchical CoT, multi-agent collaboration, self-training with DPO, and multimodal prompt retrieval have demonstrated significant enhancement in respect to interpretability, precision, efficiency, and hallucination mitigation. Third, large-scale benchmarks have a drawback: they have shown deficiencies in image-text alignment and multi-step reasoning, and the lack of standardized evaluation frameworks across diverse tasks. However, the technology is not yet ready for autonomous deployment. these approaches often introduce significant computational overhead due to iterative reasoning steps and multi-agent coordination. Such increased resource requirements may limit the feasibility for real-time clinical deployment, where rapid decision-making is critical. To ensure continued advancement in Med-VQA, future research should prioritize the development of standardized multi-step diagnostic benchmarks that better reflect real clinical reasoning workflows, including sequential imaging, temporal analysis, and multi-view interpretation. In parallel, there is a critical need to design computationally efficient reasoning frameworks that preserve high interpretability without incurring excessive overhead associated with complex approaches such as multi-agent coordination or MCTS-like strategies. Integrating external structured medical knowledge bases can play a key role in mitigating hallucinations and improving factual consistency. large-scale prospective clinical validation studies are essential to evaluate the real-world impact of Med-VQA systems on diagnostic accuracy, decision time, and patient outcomes.

## Data Availability

The original contributions presented in the study are included in the article/Supplementary Material, further inquiries can be directed to the corresponding author/s.
